# The path to a hemocompatible cardiovascular implant: Advances and challenges of current endothelialization strategies

**DOI:** 10.3389/fcvm.2022.971028

**Published:** 2022-09-14

**Authors:** Vasileios Exarchos, Ema Zacharova, Sebastian Neuber, Costanza Giampietro, Sarah E. Motta, Hristian Hinkov, Maximilian Y. Emmert, Timo Z. Nazari-Shafti

**Affiliations:** ^1^Cardiosurgical Research Group, Department of Cardiothoracic and Vascular Surgery, German Heart Center Berlin, Berlin, Germany; ^2^Translational Cardiovascular Regenerative Technologies Group, Berlin Institute of Health at Charité – Universitätsmedizin Berlin, BIH Center for Regenerative Therapies, Berlin, Germany; ^3^Department of Life Sciences, IMC University of Applied Sciences Krems, Krems an der Donau, Austria; ^4^Experimental Continuum Mechanics, Empa Swiss Federal Laboratories for Materials Science and Technology, Dübendorf, Switzerland; ^5^Department of Mechanical and Process Engineering, Institute for Mechanical Systems, ETH Zürich, Zurich, Switzerland; ^6^Institute for Regenerative Medicine, University of Zurich, Zurich, Switzerland; ^7^Clinic for Cardiovascular Surgery, Charité – Universitätsmedizin Berlin, Berlin, Germany; ^8^Department of Health Sciences and Technology, ETH Zürich, Zurich, Switzerland; ^9^Berlin Institute of Health at Charité – Universitätsmedizin Berlin, BIH Biomedical Innovation Academy, BIH Charité (Junior) (Digital) Clinician Scientist Program, Berlin, Germany

**Keywords:** cardiovascular implants, hemocompatibility, endothelialization, valves, topography

## Abstract

Cardiovascular (CV) implants are still associated with thrombogenicity due to insufficient hemocompatibility. Endothelialization of their luminal surface is a promising strategy to increase their hemocompatibility. In this review, we provide a collection of research studies and review articles aiming to summarize the recent efforts on surface modifications of CV implants, including stents, grafts, valves, and ventricular assist devises. We focus in particular on the implementation of micrometer or nanoscale surface modifications, physical characteristics of known biomaterials (such as wetness and stiffness), and surface morphological features (such as gratings, fibers, pores, and pits). We also review how biomechanical signals originating from the endothelial cell for surface interaction can be directed by topography engineering approaches toward the survival of the endothelium and its long-term adaptation. Finally, we summarize the regulatory and economic challenges that may prevent clinical implementation of endothelialized CV implants.

## Introduction

Coronary artery disease, heart valve dysfunction and heart failure are the leading causes of death worldwide (1, 2). Today, cardiovascular (CV) implants such as coronary stents, vascular grafts, bioprosthetic heart valves, and ventricular assist devices (VADs) are cornerstones in the treatment of CV disease. However, they are still associated with considerable adverse events such as thromboembolic events and infection (3).

In most cases, obstructive coronary artery disease is treated with percutaneous stent implantation or balloon angioplasty. However, both approaches increase the peri-interventional risk for endothelial damage and local inflammation, leading to restenosis due to intimal hyperplasia. To date, these adverse effect are counteracted by coating the stents with biodegradable polymers loaded with anti-proliferative drugs (e.g., sirolimus, everolimus, or paclitaxel) (4). However, local pharmacological therapy also disrupts endothelialization across the stent surface, which in turn increases the risk of late in-stent thrombosis and thus the need for a long-term inhibition of platelet aggregation (5). In patients with progressive multi-vessel coronary disease, coronary revascularization with autologous arterial grafts is the gold standard. However, in an increasingly aging and multi-morbid population, appropriate autologous arterial and venous grafts are often difficult to obtain. In addition, co-morbidities such as diabetes negatively impact graft remodeling and a significant number of patients suffer from a high rate of long-term graft failure (6). Therefore, there is an increasing clinical demand for small-diameter tissue engineered vascular grafts (TEVGs) that promote and support endothelialization at their luminal surface to avoid thrombus formation (7). In addition, widespread implantation of TEVGs could minimize wound complications associated with peripheral graft harvesting.

For patients with advanced degenerative heart valve disease, mechanical or bioprosthetic valve implantation is chosen if the native valve is unsuitable for reconstructive surgery. Patients that receive mechanical implants require lifelong anticoagulation to avoid potential thrombogenic complications (8). After implantation of a xenogeneic bioprosthetic valve, guidelines recommend anticoagulation therapy for only 3 months, because thrombotic events usually occur within the first 3 months after implantation (9). However, leaflets of bioprosthetic valves can calcify and become fibrotic over time, leading to progressive stenosis. A biocompatible fully endothelialized tissue-engineered heart valve (TEHV) may prevent early thrombus formation, chronic degeneration and generally minimizes the duration of anticoagulation therapy and associated complications.

For patients with end-stage heart failure due to coronary artery disease, heart valve dysfunction or other causes, heart transplantation and VAD support are the only available therapeutic options. However, both have their limitations and due to the increasing lack of donor organs, there will be a higher need for VAD therapy in the future (10). Unfortunately, long-term VAD implantation dramatically increases the risk of thrombosis and hemorrhagic complications due to insufficient hemocompatibility (11).

Endothelialization of vascular stents, vascular grafts, valves or even VADs may minimize the major disadvantages associated with long-term implantation of CV implants, but is still a challenging goal. For example, a plethora of reviews have reported current and evolving strategies designed to aid in the mobilization of bone marrow endothelial progenitor cells, as well as cell-specific homing, adhesion, and activation for *in situ* endothelialization (12–16). Moreover, there are numerous reviews on innovative methods that are utilized for biomaterial biofunctionalization. While many reports mainly focus on chemical modification, less attention has been paid to physical modification of biomaterial surfaces (17, 18). In addition, most of the literature focusing on physical modifications is limited regarding the effect of different nanotopographic patterns or biomaterials on basic cell functions such as adhesion, orientation, or migration (19, 20). In the present review, we look back on the last three decades and report critically on important innovative steps toward the endothelialization of CV implants (6). Furthermore, we focus on physical modification of surfaces with the aim to describe sperically the influence of surface-induced biomechanical cues on complex endothelial cell (EC) functions such as proliferation, apoptosis, inflammatory activation or even regeneration. As endothelialization strategies will target more complex CV implants in the future, we also evaluate VAD endothelialization studies and endeavor to critically comment on challenges related to the complexity and cost-effectiveness, as well as the regulatory path and clinical utility of endothelialization in CV implants.

## Function of the endothelial monolayer

ECs play an important role in hemostasis and blood-tissue barrier function, including: (i) control of vascular tone and permeability, (ii) control of coagulation and inflammation, and (iii) modulation of regenerative and anti-apoptotic pathways (21). In general, the endothelial monolayer covers the vascular luminal surface and acts as a selective barrier controlling the movement of water, proteins, metabolites and blood cells between the intravascular and interstitial space (22). Endothelial barrier functions are mainly ensured by junctional integrity, which is controlled by proteins that form the adherens junction (AJ) and tight junction (TJ) complexes, the focal adhesions (FAs) and the actin cytoskeleton. Adjacent ECs are laterally connected to their neighbors through AJs and TJs, both based on transmembrane adhesive proteins that promote homophilic interactions in a dynamic and plastic way (23, 24). Integrin-based FAs connect the basal side of ECs to the endothelial basement membrane (EBM). In the cytoplasm, the dynamic organization of actin filaments anchored to regions of cell-to-cell or cell-to-EBM contact contributes to a super-cellular mechanosensitive and mechanotransducive molecular network (25). Moreover, vascular permeability is determined by the synthesis and release of a wide range of vasoactive molecules (26). Nitric oxide (NO), the most studied vasoactive molecule is generated from L-arginine by endothelial NO synthase. NO synthesis leads to vasodilation, increases vascular permeability, inhibits platelet aggregation and modulates smooth muscle cell proliferation (22, 27). Intriguingly, NO synthesis and release, as well as inter-cellular junctions, FAs, and cytoskeletal organization are all modulated under the impact of blood flow-mediated biomechanical cues (28). The endothelium senses these signals and converts them into biological responses *via* mechanotransduction pathways to maintain its physiological function (28, 29). Under steady laminar blood flow in straight arterial segments with high shear stress acting on the endothelial luminal surface, ECs orient parallel to the flow as actin filament elongation reduces parallel intercellular stresses in favor of stability of intercellular adherent junctions and improvement of the monolayer integrity (30, 31). In addition, the physiological activation of NO synthesis and release increases blood flow to areas of the body that are deprived of oxygen and nutrients (32, 33). In contrast, impaired multidirectional turbulent blood flow at specific anatomic sites (e.g., aortic bifurcation) due to low shear stress is associated with reduced NO production and a chronic, low-grade inflammatory process. This in turn leads to a predisposition to endothelial injury and activation of the blood clotting cascade, mainly in patients with common risk factors such as hypertension, hypercholesterolemia, smoking, and diabetes mellitus (34–36).

When endothelial injury occurs, extracellular matrix (ECM) components, such as collagen, exposed to blood lead to the accumulation of platelets, red blood cells and fibrin polymerization, resulting in thrombus formation (37). In general, this coagulation process occurs *via* two pathways: (i) the extrinsic pathway, which is primarily triggered by external trauma, and (ii) the intrinsic pathway, which is triggered by damage to the inner vessel wall. The extrinsic pathway consists of the transmembrane receptor tissue factor (TF), which binds to coagulation factor VII/VIIa and initiates the coagulation cascade. The intrinsic pathway consists of plasma coagulation factors XI, IX, and VIII (37). TF is mainly expressed by perivascular cells such as adventitia fibroblasts or even vascular smooth muscle cells and circulating monocytes in response to the initiation of the coagulation cascade (38). Interestingly, mechanical injury due to excessive stretching or excessive release of circulating pro-inflammatory molecules (e.g., lipopolysaccharide, tumor necrosis factor-α, interleukin-1β, thromboxane A2) leads to EC activation and enhanced expression of TF, mainly due to nuclear translocation of the nuclear factor kappa-light-chain-enhancer of activated B cells (NF-κB). In parallel, there is a down-regulation of anticoagulant factors [e.g., thrombomodulin, tissue plasminogen activator, tissue factor pathway inhibitor (TFPI), NO synthesis] and a decrease in heparan-sulfate containing the anticoagulant glycocalyx (39). Furthermore, in activated ECs, intracellular vesicles with preformed proteins (e.g., the Weibel–Palade bodies) accumulate at the plasma membrane and release von Willebrand factor (VWF), P/E-selectin, angiopoietin-2, VCAM-1, and ICAM-1, which enhance further platelet binding and leukocyte recruitment (40). Moreover, blood cell transmigration and the release of pro-inflammatory stimuli destabilize the intercellular junctions, contributing to a progressive disruption of the endothelial barrier and the propagation of inflammation and thrombus formation (41).

Whereas permeability, monolayer integrity, and anticoagulant properties of EC monolayers are controlled by flow- or inflammation-mediated biomechanical cues as described above. Biomechanical cues derived from the interplay between FAs and the EBM appear to play a fundamental role in monolayer connectivity as well as modulation of tissue repair pathways. Intriguingly, there is a growing body of literature expressing the hypothesis that cross-regulation between AJs and FAs contributes to the maintenance of vascular barrier function (42). Furthermore, recent experimental data recognize integrins as a major player in endothelialization during angiogenesis, which regulate fundamental cellular processes including not only anchoring, polarization, and migration, but also cell proliferation, differentiation, and regeneration (43, 44). Indeed, mechanosensing can be transmitted from the cell membrane through the actin cytoskeleton to the nucleoskeleton by cell-to-surface interaction, thereby modulating gene expression associated with regenerative, tissue repair and antiapoptotic pathways (45, 46).

## Requirements for biomimetic surfaces and strategies for endothelialization

To develop a CV implant with a hemocompatible luminal surface, several key requirements must be met. The ideally biocompatible surface for endothelialization should (i) reduce or even eliminate non-specific protein adsorption (ii) enhance EC adhesion, polarization, flow-oriented elongation, and migration, leading to the establishment of an EC monolayer, (iii) prevent platelet adhesion and thrombus formation due to inflammatory EC activation and initiation of blood coagulation cascade, and (iv) activate regenerative and anti-apoptotic pathways without increasing the risk of uncontrolled proliferation and tumor growth. Considering the essential role of the EC monolayer in the homeostasis of the whole vascular wall, numerous research groups are making immense efforts to develop innovative strategies for successful endothelialization of CV implants. Two of the most common approaches for the development of CV implant surfaces aimed at mimicking and adopting the properties of ECs are physical and chemical modification. Physical modification approaches aim to engineer physical properties of biomaterials, including stiffness, wetness and surface topography, at the micro- or nanoscale topography (47). Chemical modification strategies aim to adjust chemical and biological properties of biomaterials (47). The most studied type of chemical modification is the biofunctionalization of surfaces *via* chemical adsorption, surface grafting, plasma treatment, and control of protein adsorption. Protein adsorption is the first event that occurs after implantation of a CV implant, beginning a few seconds after blood–surface interaction (48). After binding on the surface, the protein molecules (e.g., albumin and fibrinogen) show a modification of their macromolecular conformation that tends to adopt unique biochemical and physicochemical behavior (49). The progressive degradation of biomaterials, chronic recurrent infection due to biofilm formation, deleterious inflammatory, and immune responses, as well as initiation of blood coagulation cascade due to the activation of circulating blood cells and blood coagulation factors are the main effects of excessive protein deposition on the surface of biomaterials (50). Several strategies have been developed to integrate stealth properties into the surface of biomaterials that prevent protein deposition. Highly hydrophilic water-binding molecules, such as hydrophilic polysaccharides (e.g., dextran, heparin, polyacrylates, phosphorylcholine, polyethylene glycol) have been used to cover the inert blood-contacting surface, as it is well-described that the increased surface wetness is associated with reduced protein adsorption (51). In contrast, superhydrophobic micro-structured surfaces with specific geometry have been also applied to prevent highly soluble protein molecules from penetrating into the cavities of the nanostructure (52).

While numerous strategies have been focused on blocking the surface-protein interplay and initiating the blood coagulation cascade, alternative strategies aim to develop hemocompatible CV implants by promoting the endothelialization of the inert surface. Since the 1980s, the two main strategies for forming a healthy and functional endothelium on the blood-contacting surfaces of CV implants have been *in vitro* and *in vivo* endothelialization (53). For *in vitro* endothelialization, the three basic steps are: (i) EC isolation after vascularized tissue harvesting (e.g., saphenous vein or umbilical cord), (ii) *in vitro* cell expansion, and (iii) cell seeding on CV implants before implantation. *In vivo* endothelialization can be achieved by EC migration from the intact endothelium (transanastomotic ingrowth) or by adhesion, proliferation, and differentiation of circulating endothelial progenitor cells (EPCs).

While the formation of an intact EC monolayer may enhance the hemocompatibility of CV implants due to the anticoagulant properties of an intact monolayer, it is of great importance to underscore that activated endothelium due to inflammatory signals or mechanical injury, even in fully confluent monolayers, procoagulant and inflammatory properties can occur, which must be avoided in any endothelialization strategy (39). In this direction, a growing number of tissue engineering groups target to modulate complex pathways of plasmatic coagulation and inflammation strongly associated with EC activation *via*: (i) inactivation of adenosine diphosphate, GPIIb/IIIA, or VWF-dependent platelet adhesion, (ii) upregulation of anti-coagulant factors such as thrombomodulin, activated protein C, tissue factor protein inhibitor, heparin (iii) downregulation of pro-coagulant factors such as thrombin, Factor Xa, (iv) direct activation of fibrinolytic pathways based on tissue type plasminogen activator, urokinase, streptokinase and active plasmin immobilization, or (v) immobilization of immunosuppressive drugs such as everolimus, tacrolimus, and paclitaxel. (54).

Parallel, alternative experimental studies modify biological properties of CV biomaterials trying to mimic the biomechanical and biochemical native environment of ECs in favor of tissue repair, regeneration and anti-apoptotic properties (14). This includes, for example, the coating or incorporation of biomaterials with various biomolecules (e.g., NO donors) as well as growth factors [e.g., vascular endothelial growth factor (VEGF)]. These strategies have already been evaluated by several *in vitro* and animal studies as well as human clinical trials which are further discussed below (Table 1) (14).

**TABLE 1:** Past attempts to facilitate endothelialization of cardiovascular (CV) implants.

Properties/name	Luminal surface	References
**CV stents**		
Paclitaxel release TAXUS Express	Non-biodegradable coating material: poly(styrene-block-isobutylene-block-styrene) SIBS	TAXUS Clinical Trial, Stone et al. (142)
Sirolimus release CYPHER	Non-biodegradable coating materials: Parylene C, poly (ethylene-vinyl acetate), Poly(butyl methacrylate)	SIRTAX Clinical trial, Yamaji et al. (143)
Sirolimus release MiStent	Biodegradable coating material: poly(lactic-co-glycolic acid)	Dissolve clinical trial, Winter et al. (144)
Sirolimus release Ultimaster	Biodegradable coating materials: poly(D,L-lactide) and polymer light-emitting electrochemical cell	Chisari et al. (145)
Sirolimus release and EPCs attachment Combo	Biodegradable coating materials: poly(D,L-lactide) and poly(lactic-co-glycolic acid) with anti-CD34	REMEDEE clinical trial, Kerkmeijer et al. (146)
Sirolimus release Medtronic, Santa Rosa	Polymer-free	RevEvolution clinical trial, Worthley et al. (147)
Everolimus release Xience	Non-biodegradable coating materials: poly(butyl methacrylate), poly(vinylidene fluoride-co- hexafluoropropylene)	SPIRIT III clinical trial, Gada et al. (148)
Everolimus release Synergy	Biodegradable coating material: poly(D,L-lactide)	EVOLVE II clinical trial, Kereiakes et al. (149)
Zotarolimus release Resolute	Non-biodegradable coating materials: BioLinks composition of 3 polymers (C10, C19, and polyvinyl pyrrolidon)	TWENTE clinical trial, Birgelen et al. (150)
Novolimus release DESyne Nx	Non-biodegradable coating material: poly(butyl methacrylate)	EXCELLA II randomized controlled trial, Iqbal et al. (151)
Nitride oxide release TiNo stent	Polymer-free bare metal stent with titanium-NO	TIDES-ACS clinical trial, Tonino et al. (152)
Nitride oxide release Titan2 stent	Polymer-free bare metal stent with titanium-NO	TITAX clinical trial, KARJALAINEN et al. (63)
Nitride oxide release	Mussel-inspired dopamine-Cu II-coated metal stents for sustained *in situ* generation of NO	Rabbit model, Feng Zhang et al. (62)
VEGF/hepatocyte growth factor-secreting umbilical cord blood-derived mesenchymal stromal cells	Biodegradable coating material polydopamine with stem cell-secreting angiogenic growth factors	Swine model, Chang et al. (64)
Paclitaxel release	Porous composite matrix synthesized from amorphous carbon nanoparticles embedded in glassy polymeric carbon	Porcine model, Balram Bhargava et al. (153)
Paclitaxel release	Polymer-free nano-porous polymer	Porcine model, Haibo Jia et al. (154)
Sirolimus release	Polymer-free nano-porous polymer	Porcine model, Chen et al. (155)
**TEVGs**		
**Position**	**Production of TEVG**	**References**
Extracardiac total cavopulmonary conduit	Bioresorbable scaffolds of poly-l-lactide acid or poly (glycolic acid) coated with poly(l-lactic-co-ε-caprolactone) seeded with autologous bone marrow mononuclear cells	Human clinical trial in 25 pediatric patients with univentricular physiology, Tadahisa Sugiura et al. (78)
Hemodialysis conduit	Bioengineered accellular grafts produced in custom bioreactors using pulsatile circulation with cyclic radial strain followed by decellularization	Human trial in hemodialysis patients, Kirkton et al. (84)
Coronary artery bypass	Allograft saphenous veins were deendothelialized and seeded with autologous endothelial cells	Human clinical trial in 12 patients undergoing Coronary artery bypass surgery, Hermann et al. (80)
Infrarenal aortic replacement model	Hybrid grafts with poly (lactide-co-epsilon-caprolactone), collagen, and elastin loaded with heparin and VEGF	Rabbit model, Hu et al. (87)
Carotid artery bypass	Heparin and VEGF biofunctionalization of cell- free vessels based on small intestinal submucosa	Ovine model, Koobatian et al. (89)
Carotid artery bypass	Local NO delivery in decellularized xenografts derived from porcine veins promotes vascular regeneration and attenuates intimal hyperplasia and vascular calcification	Rabbit and rat model, Fei Wang et al. (88)

**Position**	**Production of TEHV**	**References**

**TEHVs**		
Pulmonary valve replacement	*In vitro* seeding of decellularized cryopreserved pulmonary allograft with autologous vascular endothelial cell	Case report of a 43 year old patient, Dohmen et al. (114)
Pulmonary valve replacement	Decellularized human pulmonary valve allografts were reseeded with peripheral EPCs isolated from human blood.	First clinical implantation of pulmonary heart valves into 2 pediatric patients, Cebotari et al. (111)
Pulmonary valve replacement	A pulmonary allograft or xenograft was decellularized, coated with fibronectin, and seeded with autologous vascular endothelial cells, isolated from a piece of forearm or saphenous vein	Human trial in 23 patients, Dohmen et al. (116)
Pulmonary valve replacement	Decellularized fresh allograft valves	Human trial in 23 patients, Cebotari et al. (115)
Pulmonary valve replacement	*In vitro* ECM production from autologous derived cells on fast biodegradable synthetic scaffolds following enzymatic decellularization	Ovine model, Driessen-Mol et al. (108)
Pulmonary valve replacement	Seeding of acellular ovine pulmonary valve scaffolds with differentiated ECs and fibroblasts after stem cell isolation from adipose tissue	Ovine model, Movileanu et al. (109)
Pulmonary valve replacement	Cell-free, slow degrading elastomeric valvular implant populated by endogenous cells	Ovine model, Kluin et al. (127)
Pulmonary valve replacement	Decellularized porcine pulmonary valves were reseeded with autologous EPCs conjugated with CD133 antibodies	Ovine model, Jordan et al. (110)
**VADs**		
**Position**	**Surface modification**	**References**
In-flow cannula	Totally sintered cannula	Tucanova et al. (137)
In-flow cannula	Totally sintered cannula	Ranjit et al. (140)
In-flow cannula	Partially sintered titanium microsphere surface	Selzman et al. (138)

## Endothelialization of cardiovascular stents

Over the last three decades, important steps toward the production of biocompatible vascular stents have been made. Since the first implantation of a bare metal stent in the mid-1980s, innovative stent technologies have contributed to the development of drug eluting stents (DESs), which hinder excessive proliferation of smooth muscle cells and prevent intima hyperplasia through the controlled release of cytostatic drugs (4). Indeed, according to numerous large-scale clinical trials, DESs significantly reduced the risk of gradual re-narrowing of the stented segment (in stent restenosis) that occurs mostly between 3 and 12 months after coronary intervention (55, 56). However, anti-proliferative agents also delay the formation of an endothelial monolayer across the stent surface and consequently increase the risk of late (after 1 month) and very late (after 1 year) stent thrombosis (5). Therefore, in addition to the gradual drug release from implants within the first 4–6 weeks, alternative surface biofunctionalization approaches were developed (57). In 2003, the first-in-man implantation of an EPC capture stent was reported. These stents attract circulating EPCs with a CD34-positive antibody-coated surface. While phase I clinical studies have demonstrated the safety and efficacy of these stents (58), data from the multi-center TRIAS HR (TRI-stent adjudication study-high risk of restenosis) clinical trial showed that EPC-capturing stents are inferior to established DESs (59). Based on these results, a biofunctionalized DES known as the COMBO stent was developed that aimed to combine the pro-endothelialization property of EPC capture technology with the abluminal elution of the anti-proliferating agent sirolimus (58). In the first human study comparing COMBO stents to paclitaxel-coated DESs, a lower rate of thrombosis was observed (60). This led the group of Caligiuri to study the role of a soluble synthetic peptide (P8RI) that acts as a CD31 agonist and increases EC adhesion, showing accelerated endothelialization upon transplantation in the swine model of female farm pigs (61).

In recent years, alternative surface biofunctionalization approaches have been also developed. In a small animal study (rabbit model), mussel-inspired dopamine-Cu II-coated metal stents improved *in situ* NO generation and decreased the risk of stent thrombosis and restenosis (62). In parallel, in 2013, the research group of Karjalainen presented the 5-year clinical outcome of the randomized control study TITAX-AMI with a total of 425 patients. Interestingly, bioactive stents coated with titanium-NO achieved greater reductions in serious cardiovascular adverse events compared to paclitaxel-eluting stents (63). In addition, in a small scale animal study (swine model), Chang et al. reported a significant pro-endothelialization effect of coronary stents seeded with vascular endothelial growth factor-secreting mesenchymal stromal cells (MSCs) (64). Large scale animal and clinical studies are needed to further investigate these endothelialization strategies.

## Endothelialization of vascular grafts

In 1954, Michael DeBakey reported the first successful distal aortic aneurysm resection and replacement in humans using a synthetic vascular graft of polyethylene terephthalate (39). Since then, large-diameter grafts made of synthetic, non-biodegradable materials have been extensively studied (65, 66). These materials have been shown to exhibit suitable mechanical properties and increased long-term durability in aortoiliac regions and other large arteries with diameters greater than 6 mm where high velocity blood flow and increased wall shear stress are encountered. However, their implementation in vessels with smaller diameters of less than 6 mm showed a significant risk of intima hyperplasia and occlusive stenosis (7). It is already known that synthetic materials are associated with high protein adsorption, which contributes to foreign body reaction and thrombus formation (67). In addition, the lack of integrin binding sites prevents attachment of circulating EPCs or expansion of adjacent cell populations such as ECs, thereby inhibiting endothelialization (68).

In the mid-1980s, Weinberg and Bell started developing TEVGs based on natural ECM components such as collagen (69). Increased biocompatibility, lower toxicity, as well as enhanced EC adhesion and proliferation were observed in small animal studies (69–72). However, the poor mechanical properties of natural polymers under the hemodynamic forces of arterial blood flow shifted the focus to the development of synthetic biodegradable polymers (e.g., polycal-prolactone, polylactic acid) (73, 74). Biodegradable polymers often act as a temporary scaffold of a blood vessel and showed promising results in many animal studies (75, 76). The group of Shin’oka published in 1998 the first attempt of TEVGs made of synthetic biodegradable materials pre-cultured *in vitro* with autologous EC as pulmonary artery autografts in sheep (74). The same group later reported the first use of TEVGs as pulmonary artery replacement after *in vitro* culture with autologous bone marrow-derived mononuclear cells in a long-term study examining 25 pediatric patients with single ventricular physiology (77, 78). Even after 7 and 11 years of follow-up, no evidence of aneurysm formation, graft rupture, graft infection, or ectopic calcification was observed. The main reason for graft failure, which occurred in a small number of patients, was graft stenosis (78, 79). These studies demonstrated for the first time that endothelialization of TEVGs is possible after implantation in the high-flow, low-pressure system of the human pulmonary artery. Whether this success can also be expected under high-pressure arterial flow in small diameter arteries still needs to be investigated. In this context, Hermann et al. presented long-term results of 12 patients undergoing coronary artery bypass surgery in 2019. Intriguingly, in de-endothelialized allograft saphenous veins seeded with autologous endothelial cells *in vitro*, graft patency was detected up to 32 months after surgery. Immunohistochemistry after death suggested that monocyte activation may lead to vessel remodeling with thickening of the vessel wall (80).

To better understand the importance of EC monolayer predisposition to the specific hemodynamical environment of small and medium diameter arteries, the research group of Niklason and Langer developed a novel bioreactor system for testing TEVGs. The bioreactor is capable of using biomimetic mechanical stimulation applying biaxial (circumferential and axial) stretching (81). First, cells seeded on the polymer scaffold produce the vessel structure according to characteristics of the native ECM during bioreactor culture. In a second step, a decellularization process removes all cells, leaving only their ECMs, onto which autologous ECs can then be seeded *in vitro* or *in vivo*, providing a ready-to-use graft solution with promising results in animal studies (82, 83). These recent achievements are expected to lead to pilot studies in humans aimed at testing the efficacy of human acellular grafts used, for instance, as arteriovenous fistulas for vascular access in patients with end-stage renal disease (84). This graft design has great potential for clinical utility but remains to be evaluated in a larger number of patients and other sites of the human circulatory system. Recently, Hermann et al. reported the first data from a small human study in which allograft saphenous veins were harvested from organ donors, cryopreserved, deendothelialized, and then seeded with autologous ECs prior to implantation in 12 patients. Immunohistochemistry revealed the presence of ECs and monocytes as well as graft patency detected up to 32 months after surgery (80). These observations highlight the importance of the ECM and natural polymer components as a promising surface for any endothelialization procedure. This motivated tissue engineering groups to focus on the development of biofunctionalized hybrid grafts with the aim to mimic the biological properties of an endothelial monolayer. In detail, coating of synthetic polymer scaffolds with natural polymers has been studied extensively to improve biocompatibility and cell adhesion (85, 86). Hu et al. investigated hybrid grafts with poly (lactide-co-epsilon-caprolactone) (PLCL), collagen, and elastin loaded with heparin and VEGF in a short-term animal study (rabbit model, follow-up of 28 days), which showed increased cell adhesion and a high EC proliferation rate (87). Similarly, Wang et al. reported enhanced vascular regeneration and inhibition of intimal hyperplasia and vascular calcification after implantation of bio-hybrid vascular grafts with local NO delivery in both rabbit and mouse models (88). In another large animal study (sheep model), an acellular tissue engineered vessel based on small intestinal submucosa functionalized with heparin and VEGF demonstrated impressive mechanical properties with concomitant successful endothelialization (89).

In conclusion, synthetic or natural non-biodegradable materials are already associated with poor clinical results due to the high risk of intima hyperplasia and occlusive stenosis or inferior mechanical properties. Since synthetic biodegradable polymers have shown encouraging results, especially in small pediatric studies, many researches are now focusing on the development of biofunctionalized hybrid grafts with biomimetic properties.

## Endothelialization of heart valves

Most commercial bioprosthetic heart valves are manufactured using glutaraldehyde-fixed xenogeneic materials, which offer an improved hemodynamic profile and reduced thrombogenicity compared to mechanical valves, thereby reducing the need for anticoagulation therapy. However, glutaraldehyde treatment does not prevent a complete antigenicity of bioprosthetic valves, contributing to a chronic immunologic response associated with calcification, progressive degeneration, and structural valve failure within 10–15 years (90). The formation of a long-living EC monolayer on the valve surface prior to implantation could minimize the risk of thrombus formation and promote tissue regeneration. It is already known that spontaneous endothelialization occurs after implantation of bioprosthetic valves, but only in a small number of patients. Additionally, if endothelialization occurs, it shows heterogenous patterns mainly close to the base of the leaflets (91). Moreover, endothelialization on surfaces pre-treated with glutaraldehyde is associated with significant cytotoxicity (92, 93). Based on *in vitro* and *in vivo* data, detoxification procedures such as treatment with L-glutamic acid promote the formation of endothelial monolayers on the surface of glutaraldehyde-preserved cardiac valves (93, 94). Therefore, from a translational perspective, less cytotoxic strategies that promote long-term EC proliferation are needed. The use of non-glutaraldehyde reagents, the application of biodegradable hydrogels, crosslinking with drug-loaded nanoparticle, the use of RGD peptides, and decellularization techniques are all tissue engineering strategies that have been developed to modify the surface of heart valves (95–101). Biomimetic acellular ECM-based TEHVs are manufactured from a polymer composite (PGA/P4HB) and an *in vitro* grown ECM, which are subsequently decellularized leaving a cell-free construct. Preclinical studies of such biomimetic valves have demonstrated their ability to undergo remodeling and recellularization, including endothelialization (102–109). Biofunctionalization of decellularized scaffolds with antibodies (e.g., CD133) to attract and capture circulating EPCs was also performed, displaying rapid *in situ* endothelialization within the first month after implantation (110, 111).

Recent clinical trials that tested human-derived pulmonary and aortic homografts showed excellent results. In detail, both the ARISE and the ESPOIR trials recently posted short term outcomes on their respective trials showing excellent outcomes for decellularized homografts. Especially the ESPOIR trial demonstrated the superiority of these grafts compared to traditional cryopreserved homografts or jugular vein conduits (112, 113). TEHVs have mostly been used as pediatric pulmonary valve replacements or as part of the Ross procedure, in which a diseased aortic valve is replaced with the patient’s own pulmonary valve (114, 115). A study of two pediatric patients implanted with decellularized pulmonary allografts showed that pre-seeding with autologous EPCs is a feasible and safe method (111). In addition, a Ross procedure study of 23 patients confirmed previous results supporting the hypothesis that heart valve decellularization and seeding with autologous vascular ECs may minimize tissue degeneration and improve valve durability (116). In a larger pulmonary valve replacement study, 38 patients were followed up to 5 years after acellular graft implantation and showed improved freedom from explanation, low gradients in echocardiographic follow-up, and adaptive growth (115). Interestingly, histological valve examination in one patient who died from non-valve related reasons revealed partial *in vivo* repopulation of the decellularized graft with autologous cells. Decellularized aortic valve allografts also appeared to be a promising alternative for aortic valve replacement.

Additionally, a variety of other materials and cell source combinations are considered for heart valve tissue engineering and have been used primarily in sheep models with varying degrees of success (117–124). Due to their versatility in mechanical, chemical and geometrical properties, synthetic bioresorbable polymers are also currently under investigation as potential starting materials for TEHV applications (125, 126). The functionality and remodeling potential of such bioresorbable polymers have been studied in several large animal models (125–128), also in combination with one-step preseeding procedures using autologous bone marrow mononuclear cells (129–133).

## Endothelialization of ventricular assist devices

Despite major technological advances, the risk of thromboembolic events on artificial surfaces that are in contact with blood limits the functionality of VADs. Various approaches have been proposed to accelerate endothelialization and thus improve VAD hemocompatibility. Texture modifications such as sintered titanium surfaces, different surface coatings (e.g., titanium nitride and heparin) and pre-seeding with engineered cells (e.g., fibroblasts) were investigated (134–136). In a small clinical study in 1987, Kurt and colleagues demonstrated how a texture modification at the luminal interface of HeartMate XVE supports endothelium formation. Since then, numerous research groups have developed innovative VADs with textured inflow cannulas (e.g., Jarvik 2000 Heart Mate II and HeartMate III) associated with low rates of apical thrombus formation and thromboembolic events (Table 1) (137–140).

However, endothelialization of the whole luminal VAD surface is very challenging, mainly due to the complex design and hemodynamic environment. To overcome these hurdles, Xi et al. recently developed a novel patterning method, harnessing the condensation and evaporation of water droplets on a curing liquid elastomer, aiming to ensure the maintenance of a protective autologous endothelium even on complex non-planar surfaces of CV implants (141). In addition, most research groups today focus on improving the physical characteristics of biomaterials such as the surface topography. Research in this field underscores the significant role of biomechanical cues generated throughout endothelialization and proposes innovative strategies to accelerate endothelialization under normal laminar flow conditions with physiological wall shear stress (WSS), or even very high supraphysiological WSS levels.

## Incorporation of biomechanical cues to enhance endothelialization

As previously described, several research groups attempted to increase the hemocompatibility of CV implants by developing biomimetic surfaces. Biological tissues in the body have a variety of physical characteristics (e.g., wetness and stiffness) as well as surface morphological features such as fibers, pores, and pits (roughness) which regulate cell behavior. For example, ECs tend to be rounder and less distributed on soft surfaces than on stiff surfaces (156). Increased pore size promotes rapid endothelialization of TEVGs, which affects not only EC adhesion, but more importantly transmural endothelialization (157). Moreover, a growing body of literature underscores that the implementation of micrometer or nanoscale surface modifications on known biomaterials enhance surface roughness and, as a result, the endothelialization of CV implants (20, 158–160). Physiologically, the formation of an EC monolayer in the native blood vessel wall requires an intact EC-EBM interplay to promote cell adhesion, cell elongation, migration and proliferation. The EC-EBM interplay starts with the formation of focal contacts. The ECs use these anchors to sense the substrate (mechanosensation) and generate a regulatory signal that is transmitted from the cytoskeleton to the nucleus (mechanotransduction) to optimize the cells’ adaptation to the surface, a phenomenon known as contact guidance (161).

Topography engineering approaches are aiming at mimicking biomechanical signals originating from the EC-EBM-ECM interplay and directing them toward accelerating endothelialization of the CV implant surface. In a systematic screening of various micro-structured substrates, Kukumberg et al. demonstrated that the surface topography either promotes or inhibits cell adhesion and cell proliferation (162). In detail, isotropic topographies such as pillars or wells resulted in lower cell density and cell proliferation compared to anisotropic topographies such as gratings. In another study by Ding et al., microgrooves on coronary stent lumen surfaces promoted EC migration and proliferation, which has also been confirmed by several research groups in the past (163–165). It was also found that ECs cultivated under physiological WSS on electrospun scaffolds are more adherent with fully aligned nanofibers than with random or semi-aligned scaffold topography (166). This observation supports the hypothesis that surface engineering of electrospun scaffolds used for CV implants may promote endothelialization and improve hemocompatibility of TEVGs or TEHVs. Nevertheless, it is still not clear how various physical properties of anisotropic features such as depth or height affect EC performance. In this direction, Potthoff et al. studied EC adhesion and migration on gratings with different dimensions under laminar flow conditions (160). As mammalian cells do not sense grooves shallower than 50 nm, gratings from 100 to 1,000 nm were examined. According to the published data, the basement membrane only contacts the top of the deep gratings (800 nm or more) and the cell membrane bridges over grooves. This led to an increase in focal adhesion kinase (FAK) activity, which ultimately contributed to improved migratory ability. Previous studies under static conditions confirmed these findings (167). Conversely, on shallow gratings (100–400 nm), the cell basement membrane interacts with both the top of the ridges and the bottom of the grooves. This led to a maturation of the FA complex and an increase in cell-surface adhesion (160). While most previous studies have focused almost exclusively on investigating the EC properties under static conditions or laminar flow with physiological WSS, Robotti et al. further investigated how 1,000 nm grids contribute to endothelialization by using a custom-designed flow bioreactor capable of reproducing supraphysiological WSS values of up to 10 Pa (168). Compared to flat surfaces, the gratings have been shown to improve monolayer integrity and support maturation of intercellular AJs, even under a WSS of 10 Pa. This data suggests that in ECs cultivated on deep gratings with 1,000 nm, FAK activation can acquire an actomyosin contractility-based pro-migratory profile that supports adhesion under flow conditions. This could potentially even promote the stability of the EC monolayer in the complex VAD hemodynamic environment in which supraphysiological WSS occurs (169). Recently, Ferrari et al. tested endothelialization on a novel hybrid membrane VAD *in vivo* (ovine animal model) and showed that a hexagonal honeycomb topography can support the generation and maintenance of a fully connected and functional endothelium (170).

While previous studies mainly focus on analyzing EC performance during endothelialization in terms of adhesion, intercellular connectivity and migratory ability, other researchers are investigating the topographical influence on factors important for EC monolayer maintenance such as proliferative ability, apoptosis, inactivation of blood coagulation and thrombus formation as well as EC regeneration. For example, Bachman et al. introduced a surface topography comprising hexagonal honeycomb shelters. EC monolayers cultivated on this topography retained their monolayer integrity even under supraphysiological loads. Intriguingly, this topography also enhanced anti-apoptotic, pro-survival and proliferative signaling pathways (171). In another experimental study on human vascular endothelial cells, different sized micro-structured surfaces with parallel micro-stripes improved NO, PGI2, TFPI, E-selectin expression, and reduced VWF secretion. Moreover, the platelet adhesion test and the whole blood clotting time test underscored the increasing anticoagulation property of elongated ECs (172). Consistent with a previous study, Huang et al. demonstrated that micropatterned and aligned nanofibrillar substrates promoted an atheroresistant EC phenotype by downregulating the expression of monocyte and platelet adhesion proteins and chemokines, supporting the hypothesis that micropatterned ECs have a unique transcriptional signature (173).

**FIGURE 1:**
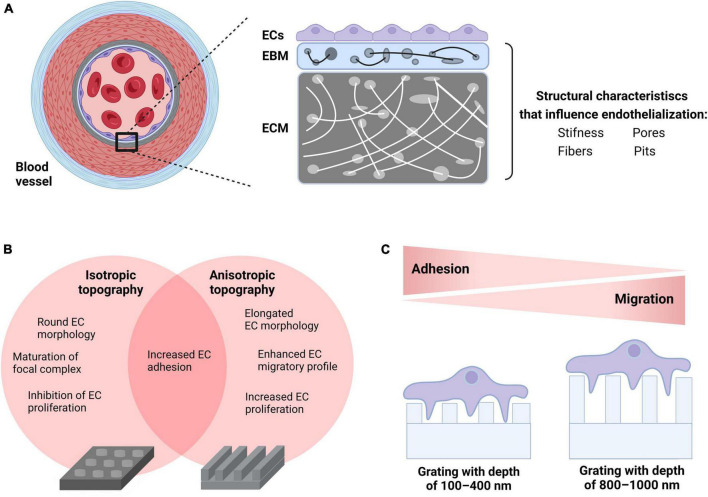
Incorporation of biomechanical cues to enhance endothelialization of CV implants. **(A)** Physiological properties of human vascular tissue such as physical (e.g., stiffness) and morphological (e.g., fibers, pores, and pits) characteristics dictate the EC-EBM-ECM interplay generating biomechanical cues that influence endothelialization. **(B)** Choice of surface geometry: ECs seeded on isotropic topographies (e.g., wells) obtain a round cellular morphology, migrate less due to the maturation of focal complexes into FAs and show low proliferation rate. In contrast, ECs seeded on anisotropic topographies such as gratings acquire an elongated cell morphology that enhances both migration and proliferation. Both surfaces increase cell adhesion under static and flow conditions. **(C)** Adjusting the surface topography: EC monolayers on shallow gratings (100–400 nm depth) interact with both the top of the ridges and the bottom of the grooves promoting FA complex maturation and enhancement of cell-surface adhesion, whereas EC monolayers on deep gratings (800–1,000 nm depth) bridge over grooves resulting in an increase in FAK activity due to enhanced phosphorylation as well as improved migratory ability. This figure was created with biorender.com.

In summary, substrate stiffness, surface geometry as well as topography may play a significant role in the early phase of endothelialization by influencing adhesion, flow-oriented cell shape elongation and migration, as well as influencing the proliferative and regenerative capacity of ECs in the long-term phase (Figure 1). *In vivo* experiments as well as large-scale clinical trials are needed to further evaluate the survival of the endothelium and its long-term adaptation to biomimetic surfaces designed for the development of fully biocompatible CV implants.

## Discussion

### Passing through the “valley of death”

Despite the success in preclinical translational research, tissue engineered products are still lacking in the clinical market (174). Currently, only 12 tissue engineering products obtained market authorization for further commercialization (175). In particular, CV implants with engineered biomimetic surfaces differ dramatically from any other biotechnological or traditional medical product. Most regulatory agencies, such as the European Medicines Agency or the Food and Drug Administration allocate CV implants to a product class with the highest safety risk classification. In addition to the safety-related engineering and physical aspects of the product, the combination with living tissue creates an additional regulatory burden. On the research and development level, it requires close cooperation between engineers, biomedical researchers and physicians. In addition to the safety and physical aspects of the product, the combination with living tissue creates an additional regulatory burden. At the research and development level, close cooperation is required between engineers, biomedical researchers and physicians. In addition, most CV implant modifications are based on existing designs, which would also require close cooperation with industry and patent holders, another major obstacle to translation and commercialization. This also explains why most bioengineered products to date are implantable valves and stents. The overall increasing demand for such products, as well as the lower “engineering” complexity, makes them easier targets for innovation and the promotion of new designs compared to more complex devices such as VADs.

### *Ex vivo* or *in vivo* endothelialization approach? The one-billion-dollar question

Endothelialization on biomaterial surfaces was originally developed by *ex vivo* cell seeding. T he fate of any *ex vivo* endothelialization strategy depends on the following factors: seeding efficiency, seeding technique and cell source (176). Seeding efficiency is mainly determined by patient characteristics (e.g., presence of cardiovascular risk factors), culture characteristics such as composition of culture media (e.g., concentration of amino acids and growth factors), as well as stimuli applied to the EC surface during *in vitro* expansion. In general, cultivation under the impact of shear stress or even circumferential dynamic forces is associated with enhanced EC performance during endothelialization (81). With regard to the seeding technique, a two-stage seeding approach has dominated in recent years. In the first step, isolated ECs are expanded *in vitro* for up to 4 weeks and in the second step, after reaching a sufficient cell number, scaffold seeding follows (177). While this approach is theoretically attractive, it also poses important regulatory issues. One the one hand, the development of good manufacturing practice-compatible standard operating procedures for harvesting and expansion of cells under ideally xeno-free culture conditions is more than necessary but an extremely time-consuming process. On the other, clean room conditions are required, which is associated with high production costs. Given the complexity of some CV devices such as VADs, seeding and maintaining an endothelial monolayer from production to implantation seems too costly and impractical. However, incorporating modifications to the luminal surfaces of CV implants may be a promising endothelialization strategy. On the one hand, physical modification of biomaterials, for example through imprintable topographies, is simple, inexpensive, safe and time-efficient as it would only add one production step during assembly of the device. However, the rapid degradation of coating biomaterials may lead to CV implant failure in the long term. On the other hand, chemical modification methods better reflect the conditions in the human body and are also characterized by a higher durability. However, they are significantly less cost-effective and very complex (178).

### Struggling to jump on the biomimetic bandwagon

The fact that cell behavior can be changed by modulating the physical and/or biochemical properties of their respective surface may open a path to a faster and less expansive regulatory road to improve endothelialization and hence hemocompatibility of CV implants. Transanastomotic endothelialization was one of the first endothelialization mechanisms identified, but it only occurs in a small area approximately 2 cm distal to the anastomosis. Furthermore, spontaneous endothelialization *in vivo* from circulating EPCs at sites of vascular ischemia and endothelial injury has been reported in animal model studies (179). However, EPCs are mainly located in the bone marrow and the number of circulating EPCs that would adhere, migrate and proliferate to form an endothelium monolayer at sites where neovascularization occurs is very limited (24). Surface modification may promote attachment, migration as well as differentiation of circulating EPCs. However, EPCs do not refer to a single cell population with a specific identity. Many researchers suggest that bone and non-bone marrow-derived cell populations can be differentiated into ECs and therefore is difficult to define, characterize and identify the “true” EPCs and their primary role in endothelial regeneration (180). Recently, several studies also supported the hypothesis that age-related vascular inflammatory disease and oxidative stress may lead to significant EPC dysfunction and EPC pool depletion, impeding the potential of endothelialization in elderly CV patients who are the main recipients of CV implants (181). Novel approaches aim to combine more than one strategy, for example by expanding EPC populations *in vitro* for vascular or coronary stent tissue engineering applications (182, 183).

In summary, despite encouraging *in vivo* data, *ex vivo* endothelialization of CV implants is associated with a significant regulatory and hence financial burden. This makes a successful bench-to-bedside-to-market translation highly challenging. Surface modifications of CV implants such as imprintable topographies may have a higher chance of bridging this so-called valley of death.

## Conclusion

Technological advances have revolutionized the design, development, and manufacturing of advanced implantable CV devices. However, due to insufficient hemocompatibility, they are still associated with devastating complications. Tissue engineering is an emerging field in contemporary health sciences aimed to optimize CV implants. This scope can be achieved through physical or biochemical surface modification of CV implants aimed to mimicking the physiological endothelial tissue function. The biochemical functionalization described above is difficult from economic and regulatory aspects. Microengineering of CV surface topography can be a promising cost-effective strategy.

## Author contributions

VE: first draft and literature research. CG: first draft. EZ and HH: literature research. SN and ME: editing. SM: literature research and editing. TN-S: first draft and editing. All authors contributed to the article and approved the submitted version.

## References

[1] Roth GA Mensah GA Johnson CO Addolorato G Ammirati E Baddour LM. Global burden of cardiovascular diseases and risk factors, 1990–2019: update from the GBD 2019 study. *J Am Coll Cardiol.* (2020) 76:2982–3021. 33309175 10.1016/j.jacc.2020.11.010 PMC7755038

[2] Bragazzi NL Zhong W Shu J Much A. Abu Lotan D Grupper A. Burden of heart failure and underlying causes in 195 countries and territories from 1990 to 2017. *Eur J Prev Cardiol.* (2021) 28:1682–90. 10.1093/eurjpc/zwaa147 33571994

[3] Zhang S Kriza C Schaller S Kolominsky-Rabas PL. Recalls of cardiac implants in the last decade: what lessons can we learn?. *PLoS One.* (2015) 10:e0125987. 10.1371/journal.pone.0125987 25962074 PMC4427435

[4] Iqbal J Gunn J Serruys PW. Coronary stents: historical development, current status and future directions. *Br Med Bull.* (2013) 106:193–211. 10.1093/bmb/ldt009 23532779

[5] Räber L Magro M Stefanini GG Kalesan B van Domburg RT Onuma Y. Very late coronary stent thrombosis of a newer-generation everolimus-eluting stent compared with early-generation drug-eluting stents: a prospective cohort study. *Circulation.* (2012) 125:1110–21. 10.1161/CIRCULATIONAHA.111.058560 22302840

[6] Pashneh-Tala S MacNeil S Claeyssens F. The tissue-engineered vascular graft-past, present, and future. *Tissue Eng Part B Rev.* (2016) 22:68–100. 10.1089/ten.teb.2015.0100 26447530 PMC4753638

[7] Durán-Rey D Crisóstomo V Sánchez-Margallo JA Sánchez-Margallo FM. Systematic Review of Tissue-Engineered Vascular Grafts. *Front Bioeng Biotechnol.* (2021) 9:771400. 10.3389/fbioe.2021.771400 34805124 PMC8595218

[8] Catterall F Ames PR Isles C. Warfarin in patients with mechanical heart valves. *BMJ.* (2020) 371:m3956. 10.1136/bmj.m3956 33060144

[9] Carlin S Eikelboom J. Advances in anticoagulation: patients with bioprosthetic heart valves. *Cardiovasc Res.* (2022) 118:e26–8. 10.1093/cvr/cvab360 35024787

[10] Garbade J Barten MJ Bittner HB Mohr FW. Heart transplantation and left ventricular assist device therapy: two comparable options in end-stage heart failure?. *Clin Cardiol.* (2013) 36:378–82. 10.1002/clc.22124 23595910 PMC6649416

[11] Potapov EV Stepanenko A Krabatsch T Hetzer R. Managing long-term complications of left ventricular assist device therapy. *Curr Opin Cardiol.* (2011) 26:237–44. 10.1097/HCO.0b013e328345af80 21460717

[12] Smith RJ Nasiri B Kann J Yergeau D Bard JE Swartz DD. Endothelialization of arterial vascular grafts by circulating monocytes. *Nat Commun.* (2020) 11:1622. 10.1038/s41467-020-15361-2 32238801 PMC7113268

[13] Melchiorri AJ Hibino N Fisher JP. Strategies and techniques to enhance the in situ endothelialization of small-diameter biodegradable polymeric vascular grafts. *Tissue Eng Part B Rev.* (2013) 19:292–307. 10.1089/ten.teb.2012.0577 23252992 PMC3690089

[14] Zhuang Y Zhang C Cheng M Huang J Liu Q Yuan G. Challenges and strategies for in situ endothelialization and long-term lumen patency of vascular grafts. *Bioact Mater.* (2021) 6:1791–809. 10.1016/j.bioactmat.2020.11.028 33336112 PMC7721596

[15] de Mel A Jell G Stevens MM Seifalian AM. Biofunctionalization of biomaterials for accelerated in situ endothelialization: a review. *Biomacromolecules.* (2008) 9:2969–79. 10.1021/bm800681k 18831592

[16] Liu T Liu S Zhang K Chen J Huang N. Endothelialization of implanted cardiovascular biomaterial surfaces: the development from in vitro to in vivo. *J Biomed Mater Res A.* (2014) 102:3754–72. 10.1002/jbm.a.35025 24243819

[17] Ren X Feng Y Guo J Wang H Li Q Yang J. Surface modification and endothelialization of biomaterials as potential scaffolds for vascular tissue engineering applications. *Chem Soc Rev.* (2015) 44:5680–742. 10.1039/C4CS00483C 26023741

[18] Jana S. Endothelialization of cardiovascular devices. *Acta Biomater.* (2019) 99:53–71. 10.1016/j.actbio.2019.08.042 31454565

[19] Bedair TM ElNaggar MA Joung YK Han DK. Recent advances to accelerate re-endothelialization for vascular stents. *J Tissue Eng.* (2017) 8:2041731417731546. 10.1177/2041731417731546 28989698 PMC5624345

[20] Zhao J Feng Y. Surface engineering of cardiovascular devices for improved hemocompatibility and rapid endothelialization. *Adv Healthc Mater.* (2020) 9:2000920. 10.1002/adhm.202000920 32833323

[21] Pober JS Sessa WC. Evolving functions of endothelial cells in inflammation. *Nat Rev Immunol.* (2007) 7:803–15. 10.1038/nri2171 17893694

[22] Rodrigues SF Granger DN. Blood cells and endothelial barrier function. *Tissue Barriers.* (2015) 3:e978720. 10.4161/21688370.2014.978720 25838983 PMC4372023

[23] Claesson-Welsh L Dejana E McDonald DM. Permeability of the endothelial barrier: identifying and reconciling controversies. *Trends Mol Med.* (2021) 27:314–31. 10.1016/j.molmed.2020.11.006 33309601 PMC8005435

[24] Dejana E. Endothelial cell–cell junctions: happy together. *Nat Rev Mol Cell Biol.* (2004) 5:261–70. 10.1038/nrm1357 15071551

[25] Lampugnani MG. Endothelial adherens junctions and the actin cytoskeleton: an ‘infinity net’?. *J Biol.* (2010) 9:16. 10.1186/jbiol232 20377920 PMC2871513

[26] Laurindo FRM Liberman M Fernandes DC Leite PF. Chapter 8 – Endothelium-dependent vasodilation: nitric oxide and other mediators. In: Da Luz PL Libby P Chagas ACP Laurindo FRM editors. *Endothelium and Cardiovascular Diseases.* London: Academic Press (2018). p. 97–113. 10.1016/B978-0-12-812348-5.00008-8

[27] Durán WN Breslin JW Sánchez FA. The NO cascade, eNOS location, and microvascular permeability. *Cardiovasc Res.* (2010) 87:254–61. 10.1093/cvr/cvq139 20462865 PMC2895543

[28] Sriram K Laughlin JG Rangamani P Tartakovsky DM. Shear-induced nitric oxide production by endothelial cells. *Biophys J.* (2016) 111:208–21. 10.1016/j.bpj.2016.05.034 27410748 PMC4944664

[29] Liu HB Zhang J Xin SY Liu C Wang CY Zhao D. Mechanosensitive properties in the endothelium and their roles in the regulation of endothelial function. *J Cardiovasc Pharmacol.* (2013) 61:461–70. 10.1097/FJC.0b013e31828c0933 23429585

[30] Choi CK Helmke BP. Short-term shear stress induces rapid actin dynamics in living endothelial cells. *Mol Cell Biomech.* (2008) 5:247–58. 20084179 PMC2806644

[31] Steward R Jr. Tambe D Hardin CC Krishnan R Fredberg JJ. Fluid shear, intercellular stress, and endothelial cell alignment. *Am J Physiol Cell Physiol.* (2015) 308:C657–64. 10.1152/ajpcell.00363.2014 25652451 PMC4398851

[32] Korenaga R Ando J Tsuboi H Yang WD Sakuma I Toyooka T. Laminar flow stimulates ATP- and shear stress-dependent nitric oxide production in cultured bovine endothelial cells. *Biochem Biophys Res Commun.* (1994) 198:213–9. 10.1006/bbrc.1994.1030 7507319

[33] Tsao PS Buitrago R Chan JR Cooke JP. Fluid flow inhibits endothelial adhesiveness. Nitric oxide and transcriptional regulation of VCAM-1. *Circulation.* (1996) 94:1682–9. 10.1161/01.CIR.94.7.1682 8840861

[34] Cybulsky MI Marsden PA. Effect of disturbed blood flow on endothelial cell gene expression. *Arterioscler Thromb Vasc Biol.* (2014) 34:1806–8. 10.1161/ATVBAHA.114.304099 25142881

[35] Ferlito S. Cardiovascular diseases and nitric oxide in humans. *Minerva Cardioangiol.* (2000) 48:379–86. 11214429

[36] Naseem KM. The role of nitric oxide in cardiovascular diseases. *Mol Aspects Med.* (2005) 26:33–65. 10.1016/j.mam.2004.09.003 15722114

[37] Yau JW Teoh H Verma S. Endothelial cell control of thrombosis. *BMC Cardiovasc Disord.* (2015) 15:130. 10.1186/s12872-015-0124-z 26481314 PMC4617895

[38] Grover SP Mackman N. Tissue Factor. *Arterioscler Thromb Vasc Biol.* (2018) 38:709–25. 10.1161/ATVBAHA.117.309846 29437578

[39] van Hinsbergh VW. Endothelium–role in regulation of coagulation and inflammation. *Semin Immunopathol.* (2012) 34:93–106. 10.1007/s00281-011-0285-5 21845431 PMC3233666

[40] Valentijn KM Sadler JE Valentijn JA Voorberg J Eikenboom J. Functional architecture of Weibel-Palade bodies. *Blood.* (2011) 117:5033–43. 10.1182/blood-2010-09-267492 21266719 PMC3109530

[41] Chistiakov DA Orekhov AN Bobryshev YV. Endothelial barrier and its abnormalities in cardiovascular disease. *Front Physiol.* (2015) 6:365. 10.3389/fphys.2015.00365 26696899 PMC4673665

[42] Pulous FE Petrich BG. Integrin-dependent regulation of the endothelial barrier. *Tissue Barriers.* (2019) 7:1685844. 10.1080/21688370.2019.1685844 31690180 PMC6866814

[43] Streuli CH. Integrins and cell-fate determination. *J Cell Sci.* (2009) 122:171–7. 10.1242/jcs.018945 19118209 PMC2714415

[44] Moreno-Layseca P Streuli CH. Signalling pathways linking integrins with cell cycle progression. *Matrix Biol.* (2014) 34:144–53. 10.1016/j.matbio.2013.10.011 24184828

[45] Martino F Perestrelo AR Vinarský V Pagliari S Forte G. Cellular mechanotransduction: from tension to function. *Front Physiol.* (2018) 9:824. 10.3389/fphys.2018.00824 30026699 PMC6041413

[46] Sun Z Guo SS Fässler R. Integrin-mediated mechanotransduction. *J Cell Biol.* (2016) 215:445–56. 10.1083/jcb.201609037 27872252 PMC5119943

[47] Raval N Kalyane D Maheshwari R Tekade RK. Chapter 17 – Surface modifications of biomaterials and their implication on biocompatibility. In: Tekade RK editor. *Biomaterials and Bionanotechnology.* London: Academic Press (2019). p. 639–74. 10.1016/B978-0-12-814427-5.00017-2

[48] Felgueiras HP Antunes JC Martins MCL Barbosa MA. 1 – Fundamentals of protein and cell interactions in biomaterials. In: Barbosa MA Martins MCL editors. *Peptides and Proteins as Biomaterials for Tissue Regeneration and Repair.* Sawston: Woodhead Publishing (2018). p. 1–27. 10.1016/B978-0-08-100803-4.00001-2

[49] Roach P Farrar D Perry CC. Interpretation of protein adsorption:? surface-induced conformational changes. *J Am Chem Soc.* (2005) 127:8168–73. 10.1021/ja042898o 15926845

[50] Jesmer AH Wylie RG. Controlling experimental parameters to improve characterization of biomaterial fouling. *Front Chem.* (2020) 8:604236. 10.3389/fchem.2020.604236 33363113 PMC7759637

[51] Kruk T Bzowska M Hinz A Szuwarzyński M. Control of specific/nonspecific protein adsorption: functionalization of polyelectrolyte multilayer films as a potential coating for biosensors. *Materials.* (2021) 14:7629. 10.3390/ma14247629 34947226 PMC8706203

[52] Zhang J Li G Man J Qu Y Guo Z Zhang S. Mechanism of anti-proteins adsorption behavior on superhydrophobic titanium surface. *Surf Coat Technol.* (2021) 421:127421. 10.1016/j.surfcoat.2021.127421

[53] Bordenave L Fernandez P Rémy-Zolghadri M Villars S Daculsi R Midy D. In vitro endothelialized ePTFE prostheses: clinical update 20 years after the first realization. *Clin Hemorheol Microcirc.* (2005) 33:227–34. 16215288

[54] Maitz MF Martins MCL Grabow N Matschegewski C Huang N Chaikof EL. The blood compatibility challenge. Part 4: surface modification for hemocompatible materials: passive and active approaches to guide blood-material interactions. *Acta Biomater.* (2019) 94:33–43. 10.1016/j.actbio.2019.06.019 31226481

[55] Kalesan B Pilgrim T Heinimann K Räber L Stefanini GG Valgimigli M. Comparison of drug-eluting stents with bare metal stents in patients with ST-segment elevation myocardial infarction. *Eur Heart J.* (2012) 33:977–87. 10.1093/eurheartj/ehs036 22362513

[56] Buccheri D Piraino D Andolina G Cortese B. Understanding and managing in-stent restenosis: a review of clinical data, from pathogenesis to treatment. *J Thorac Dis.* (2016) 8:E1150–62. 10.21037/jtd.2016.10.93 27867580 PMC5107494

[57] Lansky AJ Kereiakes DJ Baumbach A Windecker S Hussain Y Pietras C. Novel supreme drug-eluting stents with early synchronized antiproliferative drug delivery to inhibit smooth muscle cell proliferation after drug-eluting stents implantation in coronary artery disease: results of the PIONEER III randomized clinical trial. *Circulation.* (2021) 143:2143–54. 10.1161/CIRCULATIONAHA.120.052482 33820424

[58] Sethi R Lee CH. Endothelial progenitor cell capture stent: safety and effectiveness. *J Interv Cardiol.* (2012) 25:493–500. 10.1111/j.1540-8183.2012.00740.x 22612275

[59] Kerkmeijer LSM Woudstra P Klomp M Kalkman DN Varma C Koolen JJ P2798Final 5-year outcomes of the TRIAS high risk of restenosis; a multi-centre, randomized trial comparing endothelial progenitor cell capturing stent with drug-eluting stents. *Eur Heart J.* (2019) 40:ehz748.1111. 10.1093/eurheartj/ehz748.1111 21851905

[60] Woudstra P Kalkman DN den P Heijer I.B. Menown Erglis A Suryapranata H. 1-year results of the REMEDEE registry: clinical outcomes after deployment of the abluminal sirolimus-coated bioengineered (combo) stent in a multicenter, prospective all-comers registry. *JACC Cardiovasc Interv.* (2016) 9:1127–34. 27209254 10.1016/j.jcin.2016.02.052

[61] Diaz-Rodriguez S Rasser C Mesnier J Chevallier P Gallet R Choqueux C. Coronary stent CD31-mimetic coating favours endothelialization and reduces local inflammation and neointimal development in vivo. *Eur Heart J.* (2021) 42:1760–9. 10.1093/eurheartj/ehab027 33580685 PMC8106951

[62] Zhang F Zhang Q Li X Huang N Zhao X Yang Z. Mussel-inspired dopamine-Cu(II) coatings for sustained in situ generation of nitric oxide for prevention of stent thrombosis and restenosis. *Biomaterials.* (2019) 194:117–29. 10.1016/j.biomaterials.2018.12.020 30590241

[63] Tuomainen PO Ylitalo A Niemelä M Kervinen K Pietilä M Sia J. Five-year clinical outcome of titanium-nitride-oxide-coated bioactive stents versus paclitaxel-eluting stents in patients with acute myocardial infarction: long-term follow-up from the TITAX AMI trial. *Int J Cardiol.* (2013) 168:1214–9. 10.1016/j.ijcard.2012.11.060 23218575

[64] Chang H-K Kim P-H Kim DW Cho H-M Jeong MJ Kim DH. Coronary stents with inducible VEGF/HGF-secreting UCB-MSCs reduced restenosis and increased re-endothelialization in a swine model. *Exp Mol Med.* (2018) 50:1–14. 10.1038/s12276-018-0143-9 30174328 PMC6119684

[65] Boretos JW Pierce WS. Segmented polyurethane: a new elastomer for biomedical applications. *Science.* (1967) 158:1481–2. 10.1126/science.158.3807.1481 6058690

[66] Norton L Eiseman B. Replacement of portal vein during pancreatectomy for carcinoma. *Surgery.* (1975) 77:280–4. 1129701

[67] Zardeneta G Mukai H Marker V Milam SB. Protein interactions with particulate Teflon: implications for the foreign body response. *J Oral Maxillofac Surg.* (1996) 54:873–8. 10.1016/S0278-2391(96)90540-6 8676233

[68] Radke D Jia W Sharma D Fena K Wang G Goldman J. Tissue engineering at the blood-contacting surface: a review of challenges and strategies in vascular graft development. *Adv Healthc Mater.* (2018) 7:e1701461. 10.1002/adhm.201701461 29732735 PMC6105365

[69] Weinberg CB Bell E. A blood vessel model constructed from collagen and cultured vascular cells. *Science.* (1986) 231:397–400. 10.1126/science.2934816 2934816

[70] Copes F Pien N Vlierberghe S. Van Boccafoschi F Mantovani D. Collagen-based tissue engineering strategies for vascular medicine. *Front Bioeng Biotechnol.* (2019) 7:166. 10.3389/fbioe.2019.00166 31355194 PMC6639767

[71] Zavan B Vindigni V Lepidi S Iacopetti I Avruscio G Abatangelo G. Neoarteries grown in vivo using a tissue-engineered hyaluronan-based scaffold. *FASEB J.* (2008) 22:2853–61. 10.1096/fj.08-107284 18385214

[72] Swartz DD Russell JA Andreadis ST. Engineering of fibrin-based functional and implantable small-diameter blood vessels. *Am J Physiol Heart Circ Physiol.* (2005) 288:H1451–60. 10.1152/ajpheart.00479.2004 15486037

[73] McAllister T. The evolution of tissue engineered vascular grafts: from research to clinical practice. *Annu Int Conf IEEE Eng Med Biol Soc.* (2010) 2010:3589. 10.1109/IEMBS.2010.5627457 21096836

[74] Shinoka T Shum-Tim D Ma PX Tanel RE Isogai N Langer R Creation of viable pulmonary artery autografts through tissue engineering. *J Thorac Cardiovasc Surg.* (1998) 115:536–45. 10.1016/S0022-5223(98)70315-0 9535439

[75] Hoerstrup SP Cummings Mrcs I Lachat M Schoen FJ Jenni R Leschka S Functional growth in tissue-engineered living, vascular grafts: follow-up at 100 weeks in a large animal model. *Circulation.* (2006) 114:I159–66. 10.1161/CIRCULATIONAHA.105.001172 16820566

[76] Cummings I George S Kelm J Schmidt D Emmert MY Weber B. Tissue-engineered vascular graft remodeling in a growing lamb model: expression of matrix metalloproteinases. *Eur J Cardiothorac Surg.* (2012) 41:167–72. 10.1016/j.ejcts.2011.02.077 21530291 PMC3241092

[77] Shin’oka T Matsumura G Hibino N Naito Y Watanabe M Konuma T. Midterm clinical result of tissue-engineered vascular autografts seeded with autologous bone marrow cells. *J Thorac Cardiovasc Surg.* (2005) 129:1330–8. 10.1016/j.jtcvs.2004.12.047 15942574

[78] Sugiura T Matsumura G Miyamoto S Miyachi H Breuer CK Shinoka T. Tissue-engineered vascular grafts in children with congenital heart disease: intermediate term follow-up. *Semin Thorac Cardiovasc Surg.* (2018) 30:175–9. 10.1053/j.semtcvs.2018.02.002 29427773 PMC6380348

[79] Hibino N McGillicuddy E Matsumura G Ichihara Y Naito Y Breuer C Late-term results of tissue-engineered vascular grafts in humans. *J Thorac Cardiovasc Surg.* (2010) 139:431–6. 10.1016/j.jtcvs.2009.09.057 20106404

[80] Herrmann FEM Lamm P Wellmann P Milz S Hagl C Juchem G. Autologous endothelialized vein allografts in coronary artery bypass surgery – Long term results. *Biomaterials.* (2019) 212:87–97. 10.1016/j.biomaterials.2019.05.019 31108275

[81] Huang AH Lee Y-U Calle EA Boyle M Starcher BC Humphrey JD. Design and use of a novel bioreactor for regeneration of biaxially stretched tissue-engineered vessels. *Tissue Eng Part C Methods.* (2015) 21:841–51. 10.1089/ten.tec.2014.0287 25669988 PMC4523101

[82] Pellegata AF Dominioni T Ballo F Maestroni S Asnaghi MA Zerbini G. Arterial decellularized scaffolds produced using an innovative automatic system. *Cells Tissues Organs.* (2014) 200:363–73. 10.1159/000439082 26562773

[83] Quint C Arief M Muto A Dardik A Niklason LE. Allogeneic human tissue-engineered blood vessel. *J Vasc Surg.* (2012) 55:790–8. 10.1016/j.jvs.2011.07.098 22056286 PMC3505682

[84] Kirkton RD Santiago-Maysonet M Lawson JH Tente WE Dahl SLM Niklason LE Bioengineered human acellular vessels recellularize and evolve into living blood vessels after human implantation. *Sci Transl Med.* (2019) 11:eaau6934. 10.1126/scitranslmed.aau6934 30918113 PMC7557107

[85] Lu G Cui SJ Geng X Ye L Chen B Feng ZG. Design and preparation of polyurethane-collagen/heparin-conjugated polycaprolactone double-layer bionic small-diameter vascular graft and its preliminary animal tests. *Chin Med J.* (2013) 126:1310–6. 23557564

[86] Wise SG Byrom MJ Waterhouse A Bannon PG Weiss AS Ng MK. A multilayered synthetic human elastin/polycaprolactone hybrid vascular graft with tailored mechanical properties. *Acta Biomater.* (2011) 7:295–303. 10.1016/j.actbio.2010.07.022 20656079

[87] Hu YT Pan XD Zheng J Ma WG Sun LZ. In vitro and in vivo evaluation of a small-caliber coaxial electrospun vascular graft loaded with heparin and VEGF. *Int J Surg.* (2017) 44:244–9. 10.1016/j.ijsu.2017.06.077 28648794

[88] Wang F Qin K Wang K Wang H Liu Q Qian M. Nitric oxide improves regeneration and prevents calcification in bio-hybrid vascular grafts via regulation of vascular stem/progenitor cells. *Cell Rep.* (2022) 39:110981. 10.1016/j.celrep.2022.110981 35732119

[89] Koobatian MT Row S Smith RJ Jr. Koenigsknecht C Andreadis ST Swartz DD. Successful endothelialization and remodeling of a cell-free small-diameter arterial graft in a large animal model. *Biomaterials.* (2016) 76:344–58. 10.1016/j.biomaterials.2015.10.020 26561932 PMC4662921

[90] Manji RA Zhu LF Nijjar NK Rayner DC Korbutt GS Churchill TA. Glutaraldehyde-fixed bioprosthetic heart valve conduits calcify and fail from xenograft rejection. *Circulation.* (2006) 114:318–27. 10.1161/CIRCULATIONAHA.105.549311 16831988

[91] Ishihara T Ferrans VJ Jones M Boyce SW Roberts WC. Occurrence and significance of endothelial cells in implanted porcine bioprosthetic valves. *Am J Cardiol.* (1981) 48:443–54. 10.1016/0002-9149(81)90071-0 7270450

[92] Bengtsson L Radegran K Haegerstrand A. In vitro endothelialization of commercially available heart valvebioprostheses with cultured adult human cells. *Eur J Cardiothorac Surg.* (1993) 7:393–8. 10.1016/1010-7940(93)90001-R 8398184

[93] Guldner NW Jasmund I Zimmermann H Heinlein M Girndt B Meier V. Detoxification and endothelialization of glutaraldehyde-fixed bovine pericardium with titanium coating. *Circulation.* (2009) 119:1653–60. 10.1161/CIRCULATIONAHA.108.823948 19289635

[94] Lehner G Fischlein T Baretton G Murphy JG Reichart B. Endothelialized biological heart valve prostheses in the non-human primate model. *Eur J Cardiothorac Surg.* (1997) 11:498–504. 10.1016/S1010-7940(96)01096-2 9105815

[95] Yu T Yang W Zhuang W Tian Y Kong Q Chen X. A bioprosthetic heart valve cross-linked by a non-glutaraldehyde reagent with improved biocompatibility, endothelialization, anti-coagulation and anti-calcification properties. *J Mater Chem B.* (2021) 9:4031–8. 10.1039/D1TB00409C 33908590

[96] Wu B Zheng C Ding K Huang X Li M Zhang S. Cross-linking porcine pericardium by 3,4-dihydroxybenzaldehyde: a novel method to improve the biocompatibility of bioprosthetic valve. *Biomacromolecules.* (2021) 22:823–36. 10.1021/acs.biomac.0c01554 33375781

[97] Zhang S Zheng C Li M Ding K Huang X Liang X. Sodium lignosulfonate cross-linked bioprosthetic heart valve materials for enhanced cytocompatibility, improved hemocompatibility, and reduced calcification. *Compos B Eng.* (2022) 234:109669. 10.1016/j.compositesb.2022.109669

[98] Nina V Pomerantzeff P Casagrande I Chung D Benvenuti L. In vivo endothelialization of cardiac bioprostheses : conventional versus non-aldehyde preservation. *Braz J Cardiovasc Surg.* (2004) 19:144–151. 10.1590/S1678-97412004000200008

[99] Lopez-Moya M Melgar-Lesmes P. Optimizing glutaraldehyde-fixed tissue heart valves with chondroitin sulfate hydrogel for endothelialization and shielding against deterioration. *Biomacromolecule.* (2018) 19:1234–44. 10.1021/acs.biomac.8b00077 29539266 PMC6198652

[100] Hu C Luo R Wang Y. Heart valves cross-linked with erythrocyte membrane drug-loaded nanoparticles as a biomimetic strategy for anti-coagulation, anti-inflammation, anti-calcification, and endothelialization. *ACS Appl Mater Interfaces.* (2020) 12:41113–26. 10.1021/acsami.0c12688 32833422

[101] Bellis SL. Advantages of RGD peptides for directing cell association with biomaterials. *Biomaterials.* (2011) 32:4205–10. 10.1016/j.biomaterials.2011.02.029 21515168 PMC3091033

[102] Emmert MY Schmitt BA. Computational modeling guides tissue-engineered heart valve design for long-term in vivo performance in a translational sheep model. *Sci Transl Med.* (2018) 10:eaan4587. 10.1126/scitranslmed.aan4587 29743347

[103] Dijkman PE Driessen-Mol A Frese L Hoerstrup SP Baaijens FP. Decellularized homologous tissue-engineered heart valves as off-the-shelf alternatives to xeno- and homografts. *Biomaterials.* (2012) 33:4545–54. 10.1016/j.biomaterials.2012.03.015 22465337

[104] Motta SE Fioretta ES Dijkman PE Lintas V Behr L Hoerstrup SP. Development of an off-the-shelf tissue-engineered sinus valve for transcatheter pulmonary valve replacement: a proof-of-concept study. *J Cardiovasc Transl Res.* (2018) 11:182–91. 10.1007/s12265-018-9800-6 29560553

[105] Motta SE Fioretta ES Lintas V Dijkman PE Hilbe M Frese L. Geometry influences inflammatory host cell response and remodeling in tissue-engineered heart valves in-vivo. *Sci Rep.* (2020) 10:19882. 10.1038/s41598-020-76322-9 33199702 PMC7669851

[106] Motta SE Zaytseva P Fioretta ES Lintas V Breymann C Hoerstrup SP. Endothelial progenitor cell-based in vitro pre-endothelialization of human cell-derived biomimetic regenerative matrices for next-generation transcatheter heart valves applications. *Front Bioeng Biotechnol.* (2022) 10:867877. 10.3389/fbioe.2022.867877 35433657 PMC9008229

[107] Motta SE Lintas V Fioretta ES Hoerstrup SP Emmert MY. Off-the-shelf tissue engineered heart valves for in situ regeneration: current state, challenges and future directions. *Expert Rev Med Devices.* (2018) 15:35–45. 10.1080/17434440.2018.1419865 29257706

[108] Driessen-Mol A.M.Y. Emmert P.E. Dijkman L. Frese B. Sanders B. Weber Transcatheter implantation of homologous “off-the-shelf” tissue-engineered heart valves with self-repair capacity: long-term functionality and rapid in vivo remodeling in sheep. *J Am Coll Cardiol.* (2014) 63:1320–9. 10.1016/j.jacc.2013.09.082 24361320

[109] Movileanu I Harpa M Al Hussein H Harceaga L Chertes A Al Hussein H Preclinical testing of living tissue-engineered heart valves for pediatric patients, challenges and opportunities. *Front. Cardiovasc. Med.* (2021) 8:707–892. 10.3389/fcvm.2021.707892 34490371 PMC8416773

[110] Jordan JE Williams JK Lee SJ Raghavan D Atala A Yoo JJ. Bioengineered self-seeding heart valves. *J Thorac Cardiovasc Surg.* (2012) 143:201–8. 10.1016/j.jtcvs.2011.10.005 22047685

[111] Cebotari S Lichtenberg A Tudorache I Hilfiker A Mertsching H Leyh R. Clinical application of tissue engineered human heart valves using autologous progenitor cells. *Circulation.* (2006) 114:I132–7. 10.1161/CIRCULATIONAHA.105.001065 16820562

[112] Horke A Tudorache I Laufer G Andreas M Pomar JL Pereda D. Early results from a prospective, single-arm European trial on decellularized allografts for aortic valve replacement: the ARISE study and ARISE Registry data. *Eur J Cardiothorac Surg.* (2020) 58:1045–53. 10.1093/ejcts/ezaa100 32386409 PMC7577293

[113] Boethig D Horke A Hazekamp M Meyns B Rega F Van Puyvelde J. A European study on decellularized homografts for pulmonary valve replacement: initial results from the prospective ESPOIR Trial and ESPOIR Registry data†. *Eur J Cardiothorac Surg.* (2019) 56:503–9. 10.1093/ejcts/ezz054 30879050 PMC6735763

[114] Dohmen PM Lembcke A Hotz H Kivelitz D Konertz WF. Ross operation with a tissue-engineered heart valve. *Ann Thorac Surg.* (2002) 74:1438–42. 10.1016/S0003-4975(02)03881-X 12440590

[115] Cebotari S Tudorache I Ciubotaru A Boethig D Sarikouch S Goerler A Use of fresh decellularized allografts for pulmonary valve replacement may reduce the reoperation rate in children and young adults. *Circulation.* (2011) 124:S115–23. 10.1161/CIRCULATIONAHA.110.012161 21911800

[116] Dohmen PM Lembcke A Holinski S Kivelitz D Braun JP Pruss A. Mid-term clinical results using a tissue-engineered pulmonary valve to reconstruct the right ventricular outflow tract during the Ross procedure. *Ann Thorac Surg.* (2007) 84:729–36. 10.1016/j.athoracsur.2007.04.072 17720368

[117] Hoerstrup SP Sodian R Daebritz S Wang J Bacha EA Martin DP Functional living trileaflet heart valves grown in vitro. *Circulation.* (2000) 102:III44–9. 10.1161/01.CIR.102.suppl_3.III-44 11082361

[118] Syedain Z Reimer J Schmidt J Lahti M Berry J Bianco R. 6-month aortic valve implantation of an off-the-shelf tissue-engineered valve in sheep. *Biomaterials.* (2015) 73:175–84. 10.1016/j.biomaterials.2015.09.016 26409002 PMC5520964

[119] Reimer J Syedain Z Haynie B Lahti M Berry J Tranquillo R. Implantation of a Tissue-Engineered Tubular Heart Valve in Growing Lambs. *Ann Biomed Eng.* (2017) 45:439–51. 10.1007/s10439-016-1605-7 27066787 PMC5064828

[120] Syedain ZH Haynie B Johnson SL. Pediatric tri-tube valved conduits made from fibroblast-produced extracellular matrix evaluated over 52 weeks in growing lambs. *Sci Transl Med.* (2021) 13:eabb7225. 10.1126/scitranslmed.abb7225 33731437

[121] Capulli AK Emmert MY Pasqualini FS Kehl D Caliskan E Lind JU. JetValve: rapid manufacturing of biohybrid scaffolds for biomimetic heart valve replacement. *Biomaterials.* (2017) 133:229–41. 10.1016/j.biomaterials.2017.04.033 28445803 PMC5526340

[122] Fioretta ES Motta SE Lintas V Loerakker S Parker KK Baaijens FPT. Next-generation tissue-engineered heart valves with repair, remodelling and regeneration capacity. *Nat Rev Cardiol.* (2021) 18:92–116. 10.1038/s41569-020-0422-8 32908285

[123] Weber B Emmert MY Hoerstrup SP. Stem cells for heart valve regeneration. *Swiss Med Wkly.* (2012) 142:w13622. 10.4414/smw.2012.13622 22802212

[124] Fioretta ES Dijkman PE Emmert MY Hoerstrup SP. The future of heart valve replacement: recent developments and translational challenges for heart valve tissue engineering. *J Tissue Eng Regen Med.* (2018) 12:e323–35. 10.1002/term.2326 27696730

[125] Bennink G Torii S Brugmans M Cox M Svanidze O Ladich E A novel restorative pulmonary valved conduit in a chronic sheep model: mid-term hemodynamic function and histologic assessment. *J Thorac Cardiovasc Surg.* (2018) 155:2591–601.e3. 10.1016/j.jtcvs.2017.12.046 29366582

[126] Coyan GN D’Amore A Matsumura Y Pedersen DD Luketich SK Shanov V. In vivo functional assessment of a novel degradable metal and elastomeric scaffold-based tissue engineered heart valve. *J Thorac Cardiovasc Surg.* (2019) 157:1809–16. 10.1016/j.jtcvs.2018.09.128 30578064

[127] Kluin J Talacua H Smits AI Emmert MY Brugmans MC Fioretta ES. In situ heart valve tissue engineering using a bioresorbable elastomeric implant – From material design to 12 months follow-up in sheep. *Biomaterials.* (2017) 125:101–17. 10.1016/j.biomaterials.2017.02.007 28253994

[128] Soliman OI Miyazaki Y Abdelghani M Brugmans M Witsenburg M Onuma Y. Midterm performance of a novel restorative pulmonary valved conduit: preclinical results. *EuroIntervention.* (2017) 13:e1418–27. 10.4244/EIJ-D-17-00553 28829747

[129] Weber B Scherman J Emmert MY Gruenenfelder J Verbeek R Bracher M. Injectable living marrow stromal cell-based autologous tissue engineered heart valves: first experiences with a one-step intervention in primates. *Eur Heart J.* (2011) 32:2830–40. 10.1093/eurheartj/ehr059 21415068

[130] Emmert MY Weber B Behr L Frauenfelder T Brokopp CE Grünenfelder J. Transapical aortic implantation of autologous marrow stromal cell-based tissue-engineered heart valves: first experiences in the systemic circulation. *JACC Cardiovasc Interv.* (2011) 4:822–3. 10.1016/j.jcin.2011.02.020 21777893

[131] Emmert MY Weber B Wolint P Behr L Sammut S Frauenfelder T. Stem cell-based transcatheter aortic valve implantation: first experiences in a pre-clinical model. *JACC Cardiovasc Interv.* (2012) 5:874–83. 10.1016/j.jcin.2012.04.010 22917460

[132] Emmert MY Weber B Behr L Sammut S Frauenfelder T Wolint P. Transcatheter aortic valve implantation using anatomically oriented, marrow stromal cell-based, stented, tissue-engineered heart valves: technical considerations and implications for translational cell-based heart valve concepts. *Eur J Cardiothorac Surg.* (2014) 45:61–8. 10.1093/ejcts/ezt243 23657551

[133] Fioretta ES Lintas V Mallone A Motta SE von Boehmer L Dijkman PE. Differential leaflet remodeling of bone marrow cell pre-seeded versus nonseeded bioresorbable transcatheter pulmonary valve replacements. *JACC Basic Transl Sci.* (2020) 5:15–31. 10.1016/j.jacbts.2019.09.008 32043018 PMC7000873

[134] Noviani M Jamiolkowski RM Grenet JE Lin Q Carlon TA Qi L. Point-of-care rapid-seeding ventricular assist device with blood-derived endothelial cells to create a living antithrombotic coating. *ASAIO J.* (2016) 62:447–53. 10.1097/MAT.0000000000000351 26809085 PMC4925231

[135] Sin DC Kei HL Miao X. Surface coatings for ventricular assist devices. *Expert Rev Med Devices.* (2009) 6:51–60. 10.1586/17434440.6.1.51 19105780

[136] Scott-Burden T Tock CL Bosely JP Clubb FJ Parnis SM Schwarz JJ Nonthrombogenic, adhesive cellular lining for left ventricular assist devices. *Circulation.* (1998) 98:Ii339–45. 9852924

[137] Tucanova Z Ivak P Konarik M Szarszoi O Fabian O Melenovsky V Systematic evaluation of heartmate 3 inflow cannula at transplant and the association with reduced anticoagulation. *J Heart Lung Transplant.* (2022) 41:S487. 10.1016/j.healun.2022.01.1231

[138] Selzman CH Koliopoulou A Glotzbach JP McKellar SH. evolutionary improvements in the Jarvik 2000 left ventricular assist device. *ASAIO J.* (2018) 64:827–30. 10.1097/MAT.0000000000000743 29324511

[139] Kurtyka P Kustosz R Kaczmarek M Gonsior M Tokarska K. Surface modifications for inflow cannulas of ventricular assist devices – comparison of latest solutions. *Eng Biomater.* (2019) 22:17–23.

[140] John R Kamdar F Liao K Colvin-Adams M Miller L Joyce L. Low thromboembolic risk for patients with the Heartmate II left ventricular assist device. *J Thorac Cardiovasc Surg.* (2008) 136:1318–23. 10.1016/j.jtcvs.2007.12.077 19026822

[141] Wu X Moimas S Hopf R Giampietro C Kourouklis A Falk V. A free-form patterning method enabling endothelialization under dynamic flow. *Biomaterials.* (2021) 273:120816. 10.1016/j.biomaterials.2021.120816 33895492

[142] Stone GW Ellis SG Colombo A Grube E Popma JJ Uchida T. Long-term safety and efficacy of paclitaxel-eluting stents final 5-year analysis from the TAXUS clinical trial program. *JACC Cardiovasc Interv.* (2011) 4:530–42. 10.1016/j.jcin.2011.03.005 21596326

[143] Yamaji K Räber L Zanchin T Spitzer E Zanchin C Pilgrim T. Ten-year clinical outcomes of first-generation drug-eluting stents: the Sirolimus-Eluting vs. paclitaxel-eluting stents for coronary revascularization (SIRTAX) VERY LATE trial. *Eur Heart J.* (2016) 37:3386–95. 10.1093/eurheartj/ehw343 27578808

[144] de Winter RJ Katagiri Y Asano T Milewski KP Lurz P Buszman P. A sirolimus-eluting bioabsorbable polymer-coated stent (MiStent) versus an everolimus-eluting durable polymer stent (Xience) after percutaneous coronary intervention (DESSOLVE III): a randomised, single-blind, multicentre, non-inferiority, phase 3 trial. *Lancet.* (2018) 391:431–40. 10.1016/S0140-6736(17)33103-3 29203070

[145] Chisari A Pistritto AM Piccolo R Manna A. La Danzi GB. The ultimaster biodegradable-polymer sirolimus-eluting stent: an updated review of clinical evidence. *Int J Mol Sci.* (2016) 17:1490. 10.3390/ijms17091490 27608017 PMC5037768

[146] Kerkmeijer LSM Chandrasekhar J. Final five-year results of the REMEDEE registry: real-world experience with the dual-therapy COMBO stent. *Catheter Cardiovasc Interv.* (2021) 98:503–10. 10.1002/ccd.29305 33029937 PMC8518525

[147] Worthley SG Abizaid A Kirtane AJ Simon DI Windecker S Brar S. First-in-human evaluation of a novel polymer-free drug-filled stent: angiographic, IVUS, OCT, and clinical outcomes from the revelution study. *JACC Cardiovasc Interv.* (2017) 10:147–56. 28104208 10.1016/j.jcin.2016.10.020

[148] Gada H Kirtane AJ Newman W Sanz M Hermiller JB Mahaffey KW. 5-year results of a randomized comparison of XIENCE V everolimus-eluting and TAXUS paclitaxel-eluting stents: final results from the SPIRIT III Trial (clinical evaluation of the XIENCE V everolimus eluting coronary stent system in the treatment of patients with de novo native coronary artery lesions). *JACC Cardiovasc Interv.* (2013) 6:1263–6. 10.1016/j.jcin.2013.07.009 24239202

[149] Kereiakes DJ Windecker S Jobe RL Mehta SR Sarembock IJ Feldman RL. Clinical outcomes following implantation of thin-strut, bioabsorbable polymer-coated, everolimus-eluting SYNERGY stents. *Circ Cardiovasc Interv.* (2019) 12:e008152. 10.1161/CIRCINTERVENTIONS.119.008152 31451014

[150] von Birgelen C van der Heijden LC Basalus MWZ Kok MM Sen H Louwerenburg HW. Five-year outcome after implantation of zotarolimus- and everolimus-eluting stents in randomized trial participants and nonenrolled eligible patients: a secondary analysis of a randomized clinical trial. *JAMA Cardiol.* (2017) 2:268–76. 10.1001/jamacardio.2016.5190 28114618

[151] Iqbal J Verheye S Abizaid A Ormiston J de Vries T Morrison L. DESyne novolimus-eluting coronary stent is superior to Endeavor zotarolimus-eluting coronary stent at five-year follow-up: final results of the multicentre EXCELLA II randomised controlled trial. *EuroIntervention.* (2016) 12:e1336–42. 10.4244/EIJY15M10_04 26465374

[152] Tonino PAL Pijls NHJ Collet C Nammas W Van der Heyden J Romppanen H. Titanium-nitride-oxide-coated versus everolimus-eluting stents in acute coronary syndrome: the randomized TIDES-ACS trial. *JACC Cardiovasc Interv.* (2020) 13:1697–705. 10.1016/j.jcin.2020.04.021 32703593

[153] Bhargava B Reddy NK Karthikeyan G Raju R Mishra S Singh S. A novel paclitaxel-eluting porous carbon-carbon nanoparticle coated, nonpolymeric cobalt-chromium stent: evaluation in a porcine model. *Catheter Cardiovasc Interv.* (2006) 67:698–702. 10.1002/ccd.20698 16575925

[154] Jia H Liu H Kong J Hou J Wu J Zhang M. A novel polymer-free paclitaxel-eluting stent with a nanoporous surface for rapid endothelialization and inhibition of intimal hyperplasia: comparison with a polymer-based sirolimus-eluting stent and bare metal stent in a porcine model. *J Biomed Mater Res A.* (2011) 98:629–37. 10.1002/jbm.a.33151 21732525

[155] Chen M Zheng B Wu Z Peng HY Wang XG Zhang B. Efficacy and safety of a novel nano-porous polymer-free sirolimus-eluting stent in pigs. *Chin Med J.* (2013) 126:4731–5. 24342320

[156] Yi B Shen Y Tang H Wang X Zhang Y. Stiffness of the aligned fibers affects structural and functional integrity of the oriented endothelial cells. *Acta Biomater.* (2020) 108:237–49. 10.1016/j.actbio.2020.03.022 32205213

[157] O’Brien FJ Harley BA Waller MA Yannas IV Gibson LJ Prendergast PJ. The effect of pore size on permeability and cell attachment in collagen scaffolds for tissue engineering. *Technol Health Care.* (2007) 15:3–17. 10.3233/THC-2007-15102 17264409

[158] Stolberg S McCloskey KE. Can shear stress direct stem cell fate?. *Biotechnol Prog.* (2009) 25:10–9. 10.1002/btpr.124 19197983

[159] Karki P Birukova AA. Substrate stiffness-dependent exacerbation of endothelial permeability and inflammation: mechanisms and potential implications in ALI and PH (2017 Grover Conference Series). *Pulm Circ.* (2018) 8:2045894018773044. 10.1177/2045894018773044 29714090 PMC5987909

[160] Potthoff E Franco D D’Alessandro V Starck C Falk V Zambelli T. Toward a rational design of surface textures promoting endothelialization. *Nano Lett.* (2014) 14:1069–79. 10.1021/nl4047398 24428164

[161] Almonacid Suarez AM van der Ham I Brinker MGL van Rijn P Harmsen MC. Topography-driven alterations in endothelial cell phenotype and contact guidance. *Heliyon.* (2020) 6:e04329. 10.1016/j.heliyon.2020.e04329 32637708 PMC7330714

[162] Kukumberg M Yao Y Goh SH Neo DJ Yao JY Yim EK. Evaluation of the topographical influence on the cellular behavior of human umbilical vein endothelial cells. *Adv Biosyst.* (2018) 2:1700217. 10.1002/adbi.201700217 30766915 PMC6370334

[163] Ding Y Yang Z Bi CW Yang M Xu SL Lu X. Directing vascular cell selectivity and hemocompatibility on patterned platforms featuring variable topographic geometry and size. *ACS Appl Mater Interfaces.* (2014) 6:12062–70. 10.1021/am502692k 25039647

[164] Sprague EA Tio F Ahmed SH Granada JF Bailey SR. Impact of parallel micro-engineered stent grooves on endothelial cell migration, proliferation, and function. *Circ Cardiovasc Interv.* (2012) 5:499–507. 10.1161/CIRCINTERVENTIONS.111.967901 22763346

[165] Tan J Bai J Yan Z. An aligned patterned biomimetic elastic membrane has a potential as vascular tissue engineering material. *Front Bioeng Biotechnol.* (2020) 8:704. 10.3389/fbioe.2020.00704 32695769 PMC7338373

[166] Whited BM Rylander MN. The influence of electrospun scaffold topography on endothelial cell morphology, alignment, and adhesion in response to fluid flow. *Biotechnol Bioeng.* (2014) 111:184–95. 10.1002/bit.24995 23842728 PMC3878428

[167] Franco D Klingauf M Bednarzik M Cecchini M Kurtcuoglu V Gobrecht J. Control of initial endothelial spreading by topographic activation of focal adhesion kinase. *Soft Matter.* (2011) 7:7313–24. 10.1039/c1sm05191a

[168] Robotti F Franco D Bänninger L Wyler J Starck CT Falk V. The influence of surface micro-structure on endothelialization under supraphysiological wall shear stress. *Biomaterials.* (2014) 35:8479–86. 10.1016/j.biomaterials.2014.06.046 25017097

[169] Mierke CT Fischer T Puder S Kunschmann T Soetje B Ziegler WH. Focal adhesion kinase activity is required for actomyosin contractility-based invasion of cells into dense 3D matrices. *Sci Rep.* (2017) 7:42780. 10.1038/srep42780 28202937 PMC5311912

[170] Ferrari A Giampietro C Bachmann B Bernardi L Bezuidenhhout D Ermanni P. A novel hybrid membrane VAD as first step toward hemocompatible blood propulsion. *Ann Biomed Eng.* (2021) 49:716–31. 10.1007/s10439-020-02590-1 32901382 PMC7851026

[171] Bachmann BJ Giampietro C Bayram A Stefopoulos G Michos C Graeber G. Honeycomb-structured metasurfaces for the adaptive nesting of endothelial cells under hemodynamic loads. *Biomater Sci.* (2018) 6:2726–37. 10.1039/C8BM00660A 30159552

[172] Li J Zhang K Yang P Qin W Li G Zhao A. Human vascular endothelial cell morphology and functional cytokine secretion influenced by different size of HA micro-pattern on titanium substrate. *Colloids Surf B Biointerfaces.* (2013) 110:199–207. 10.1016/j.colsurfb.2013.04.048 23732795

[173] Huang NF Lai ES Ribeiro AJ Pan S Pruitt BL Fuller GG. Spatial patterning of endothelium modulates cell morphology, adhesiveness and transcriptional signature. *Biomaterials.* (2013) 34:2928–37. 10.1016/j.biomaterials.2013.01.017 23357369 PMC3581686

[174] O’Donnell BT Ives CJ Mohiuddin OA Bunnell BA. Beyond the present constraints that prevent a wide spread of tissue engineering and regenerative medicine approaches. *Front Bioeng Biotechnol.* (2019) 7:95. 10.3389/fbioe.2019.00095 31134194 PMC6514054

[175] Oberweis CV Marchal JA López-Ruiz E Gálvez-Martín P. A worldwide overview of regulatory frameworks for tissue-based products. *Tissue Eng Part B Rev.* (2020) 26:181–96. 10.1089/ten.teb.2019.0315 31910099

[176] Sánchez PF Brey EM Briceńo JC. Endothelialization mechanisms in vascular grafts. *J Tissue Eng Regen Med.* (2018) 12:2164–78. 10.1002/term.2747 30079631

[177] de Valence S Tille JC Mugnai D Mrowczynski W Gurny R Möller M. Long term performance of polycaprolactone vascular grafts in a rat abdominal aorta replacement model. *Biomaterials.* (2012) 33:38–47. 10.1016/j.biomaterials.2011.09.024 21940044

[178] Kurpanik R Stodolak-Zych E. Chemical and physical modifications of electrospun fibers as a method to stimulate tissue regeneration – minireview. *Eng Biomater.* (2021) 159:31–41.

[179] Asahara T Murohara T Sullivan A Silver M Zee RVD Li T. Isolation of putative progenitor endothelial cells for angiogenesis. *Science.* (1997) 275:964–6. 10.1126/science.275.5302.964 9020076

[180] Urbich C Dimmeler S. Endothelial progenitor cells. *Circ Res.* (2004) 95:343–53. 10.1161/01.RES.0000137877.89448.78 15321944

[181] Balistreri CR Buffa S Pisano C Lio D Ruvolo G Mazzesi G. Are endothelial progenitor cells the real solution for cardiovascular diseases? focus on controversies and perspectives. *Biomed Res Int.* (2015) 2015:835934. 10.1155/2015/835934 26509164 PMC4609774

[182] Tsukada J Wolf F Vogt F Schaaps N Thoröe-Boveleth S Keijdener H. Development of in vitro endothelialized drug-eluting stent using human peripheral blood-derived endothelial progenitor cells. *J Tissue Eng Regen Med.* (2020) 14:1415–27. 10.1002/term.3107 32668066

[183] Melchiorri AJ Bracaglia LG Kimerer LK Hibino N Fisher JP. In vitro endothelialization of biodegradable vascular grafts via endothelial progenitor cell seeding and maturation in a tubular perfusion system bioreactor. *Tissue Eng Part C Methods.* (2016) 22:663–70. 10.1089/ten.tec.2015.0562 27206552 PMC4943466

